# Cyclic stretch induced IL-33 production through HMGB1/TLR-4 signaling pathway in murine respiratory epithelial cells

**DOI:** 10.1371/journal.pone.0184770

**Published:** 2017-09-12

**Authors:** Jing Chang, Yuefeng Xia, Karla Wasserloos, Meihong Deng, Kory J. Blose, David A. Vorp, Heth R. Turnquist, Timothy R. Billiar, Bruce A. Pitt, Ma-Zhong Zhang, Li-Ming Zhang

**Affiliations:** 1 Department of Anesthesiology, Shanghai Children’s Medical Center, Shanghai Jiao-Tong University School of Medicine, Shanghai, China; 2 Department of Environmental and Occupational Health, University of Pittsburgh Graduate School Public Health, Pittsburgh, Pennsylvania, United States of America; 3 Department of Anesthesiology, University of Pittsburgh School of Medicine, Pittsburgh, Pennsylvania, United States of America; 4 Department of Anesthesiology, Hunan Cancer Hospital, Xiangya School of Medicine, Central South University, Hunan, China; 5 Department of Surgery, University of Pittsburgh School of Medicine, Pittsburgh, Pennsylvania, United States of America; 6 Department of Bioengineering, University of Pittsburgh, Pittsburgh, Pennsylvania, United States of America; 7 Department of Cardiothoracic Surgery, University of Pittsburgh, Pittsburgh, Pennsylvania, United States of America; Universitatsklinikum Freiburg, GERMANY

## Abstract

Interleukin 33 (IL-33), an inflammatory and mechanically responsive cytokine, is an important component of a TLR4-dependent innate immune process in mucosal epithelium. Although TLR4 also plays a role in sensing biomechanical stretch, a pathway of stretch-induced TLR4-dependent IL-33 biosynthesis has not been revealed. In the current study, we show that short term (6 h) cyclic stretch (CS) of cultured murine respiratory epithelial cells (MLE-12) increased intracellular IL-33 expression in a TLR4 dependent fashion. There was no detectable IL-33 in conditioned media in this interval. CS, however, increased release of the notable alarmin, HMGB1, and a neutralizing antibody (2G7) to HMGB1 completely abolished the CS mediated increase in IL-33. rHMGB1 increased IL-33 synthesis and this was partially abrogated by silencing TLR4 suggesting additional receptors for HMGB1 are involved in its regulation of IL-33. Collectively, these data reveal a HMGB1/TLR4/IL-33 pathway in the response of respiratory epithelium to mechanical stretch.

## Introduction

Mechanical ventilation, a common requisite component of intensive (to reduce work of breathing) and perioperative (for adequate gas exchange and the delivery of volatile anesthetics) care is well known to cause an iatrogenic syndrome of ventilator induced lung injury (VILI) [1]. Physical forces (e.g. overdistension) accounting for VILI may be transduced into biological forces (production of pro-inflammatory intracellular mediators and injurious pathways) via cellular mechanisms that are poorly understood. In the complex setting of intact mice, Toll-like receptor 4 (TLR4) has been shown by several groups to be critical in the pathophysiology of VILI [2–5]. Stretching isolated cardiomyocytes [6] and respiratory epithelium [7] potentially activated TLR4 by increasing its overall or surface expression, respectively. Stretching primary alveolar type II cells [8] or murine lung epithelial (MLE-12) cells [7] after activation of TLR4 with lipopolysaccharide (LPS) did not exacerbate innate immune response or decreased production of inflammatory cytokines and procoagulant molecules, respectively. In contrast, TLR4 was essential for formation of inflammasome and production of interleukin-1β (IL-1β) in isolated stretched alveolar macrophages [9].

We sought to further investigate the contributory role of TLR4 in the context of interleukin-33 (IL-33) biosynthesis in stretched cultured MLE-12 cells. Since its original discovery [10] as the functional ligand for ST2, an IL-1 receptor family member, IL-33 has been shown to act as an alarmin [11] and a mechanically responsive cytokine in cardiomyocytes and fibroblasts [12, 13]. IL-33 is expressed in the lung [10] and in pulmonary endothelium [14] and intestinal epithelium [15]. The increase in immunoreactive IL-33 in the alveolar wall of mechanically ventilated rats [16] suggests a role for IL-33 in VILI although isolated type II cells in short term culture from intact mice subjected to high tidal volume mechanical ventilation did not show an increase in IL-33 [17]. A TLR4-dependent IL-33 signaling pathway involving ST2 signaling/Th2 pathways in allergic inflammation in mice was recently reported [18, 19]. We recently reviewed IL-33 signaling in lung injury [20] and reported that IL-33 drives acute lung injury after systemic injury [21]. However, the link between IL-33 and TLR4 in non-infectious, non-allergic biosensing to mechanical stretch remains unclear.

High mobility group box 1 (HMGB1) is an abundant nonhistone nuclear protein ubiquitously expressed in resting cells [22]. Like IL-33, it is thought to be released from necrotic cells to the extracellular space mediating inflammation and acting as an alarmin. A number of cell surface receptors are critical in such activity including receptor for advanced glycation end-products (RAGE) and TLR4. HMGB1 is a critical molecule in a number of forms of acute lung injury including VILI as HMGB1 is increased with cyclic stretch and LPS exposure in A549 cells [23]. A cardiomyocyte HMGB1/fibroblast TLR4/IL-33 axis contributes to diabetic cardiomyopathy in mice [24].

In the current study, we stretched (~18% elongation) isolated cultured MLE-12 cells on a flexible membrane in cyclic (1 Hz) short term fashion and noted a TLR4 dependent increase in intracellular IL-33 and extracellular HMGB1 at 6 h. CS-induced increase in IL-33 was abrogated by neutralizing antibodies to HMGB1 placing HMGB1 upstream of TLR4 mediated IL-33 biosynthesis.

## Materials and methods

### Cell culture

Mouse lung epithelial cells (MLE-12) were cultured in DMEM/F-12 medium (ATCC) supplemented with 5 μg/ml insulin, 10 μg/ml transferrin, 30 nM sodium selenite, 10 nM hydrocortisone, 10 nM beta-estradiol, 2 mM L-glutamine, 10 mM HEPES, and 10% fetal bovine serum (Sigma-Aldrich, St. Louis, MO). Cells were cultured at 37°C in 5% CO_2_ and were subcultured continuously (2×/wk) for a maximum of 32 sub-passages. In some experiments, LPS (100 ng/ml) was added to serum free medium (12 h). Ultrapure LPS (*Escherichia coli* 0111:B4) was from List Biological Laboratories (Vandell way, CA) and is reported to be free of contaminating proteins and to selectively activate TLR4 [25]. HMGB1 neutralizing antibody, 2G7 [15, 26, 27], was kindly provided by Kevin J. Tracey (Feinstein Institute of Medical Technology) and 10μg/ml HMGB1 neutralizing antibody was put into media before stretch. Recombinant (r)HMGB1 was from Santa Cruz. Cells were exposed before and during CS (or in control conditions) to 3 μg/ml HMGB1.

### Cell stretching protocol

MLE-12 cells were placed on the central area (1.5 cm diameter) of fibronectin-coated silicon membranes (Bioflex; Flexcell International, Hillsborough, NC; coated additionally with 150 M bovine fibronectin for at least 3 h at 4°C) of six-well plates at density of 0.35~0.4×10^6^ cells per well. Density of 0.2–0.25×10^6^ cells per well was used when transfecting the cells with TLR-4 siRNA before stretching. After 24 h of adherence, medium was replaced by fresh DMEM/F-12 medium. These plates were used for experiments. Medium was replaced by serum free media 12 h before stretching.

MLE-12 cells on Bioflex plates were exposed to stretch using the FX 4000T Flexercell Tension Plus system (Flexcell International) as we recently described [28]. Stretching patterns were defined by frequency and elongation and were either static (~18% elongation) or cyclic (CS: 1Hz, ~18% elongation). The plates were deformed through regulated air vacuum supplied to the bottom of the plate causing the membrane to stretch across a loading post [28]. Cells and media were collected at specific time point. Membrane distension was calibrated and monitored during the experiment. A subgroup of MLE-12 cells were placed in the identical media and subcultured on stretching plates but not subjected to stretch and served as controls. Stretching groups consisted of 3 replicate wells and experiments repeated on at least 3 separate occasions.

### Flow cytometry

Stretched and non-stretched (control) cells were rinsed in PBS, trypsinized, and centrifuged at 1,500 rpm for 5 min. The cell pellet was resuspended in 300 μl binding buffer and supplemented with 3 μl of FITC-annexin-V and 3 μl of propidium iodide (PI) and incubated at room temperature for 15 min in the dark. Flow cytometric analysis was performed using a FACSCanto (BD Biosciences, San Jose, CA). For each sample 10,000 events were recorded and analyzed.

### TLR4 siRNA

MLE-12 cells were transfected with 50 nM TLR4-specific siRNA or nonspecific scrambled siRNA as a control (Invitrogen, Carlsbad, CA) using Lipofectamine 2000 (Invitrogen, Carlsbad, CA) according to the manufacturer’s protocol and then incubated at 37°C in a 5% CO_2_ incubator for at least 48 h before stretching. The efficacy of knockdown was determined by western blot.

### ELISA and luminex

Culture media and lysates of cells were prepared in order to quantify the levels of cytokines. Cytokine concentrations were measured using specific ELISA assays for IL-33, IL-6, (R&D Systems, Minneapolis, MN, USA), HMGB1 (Tecan Trading AG, Switzerland). The assay procedures were performed according to manufacturer. Luminex was also used to detect cytokines IL-6, IL-33 (and other cytokines not reported) by mouse Th17 Magnetic Bead Panel (EMD Millipore Corporation, MA, USA). The same amount of protein (samples were quantitated to 1 μg/μl in a total of 50 μg) were loaded for detection.

### Western blot

Cell extracts were lysed on ice with radio immunoprecipitation assay-buffer (Thermo Fisher Scientific, Rockford, IL, USA). Nuclear and cytoplasm protein fractions were isolated with NE-PER nuclear and cytoplasmic protein extraction reagents (Thermo Fisher Scientific, Rockford, IL, USA). Protein concentrations were determined by microplate BCA protein assay kit-reducing agent compatible (Thermo Fisher Scientific, Rockford, IL, USA). For Western blotting, 30 μg of protein per lane were loaded on to NuPAGE^TM^ 4–12% Bis-Tris gels (Invitrogen, Carlsbad, CA).

Primary polyclonal antibody against mouse IL-33 was purchased from R&D Systems, (Minneapolis, MN, USA), Toll-like receptor 4 monoclonal antibody (mouse specific) was purchased from Cell Signaling (Danvers, MA, USA). HMGB1 antibody was purchased from Abcam (Cambridge, MA, USA), NF-κB p65 monoclonal antibody was purchased from Cell Signaling (Danvers, MA, USA). The biotinylated secondary antibody was purchased from Santa Cruz Biotechnology (Dallas, TX, USA).

The bands were detected using Plus-ECL enhanced chemiluminescence kit (PerkinElmer, MA, USA). Membranes were stripped and reprobed for β-actin or LaminB (Sigma Aldrich, MO, USA) that served as a loading control.

### Statistical analysis

Data are mean ± SD from 3–5 separate experiments. Statistical significance was defined as P < 0.05 and was determined by either two-way or one-way ANOVA, followed by Tukey’s post test, using Graphpad Prism ver. 7.0 (GraphPad Software, San Diego, CA).

## Results

### Cyclic stretch (CS) increases IL-33 expression in a TLR-4 dependent fashion in murine respiratory epithelium

**Fig 1:**
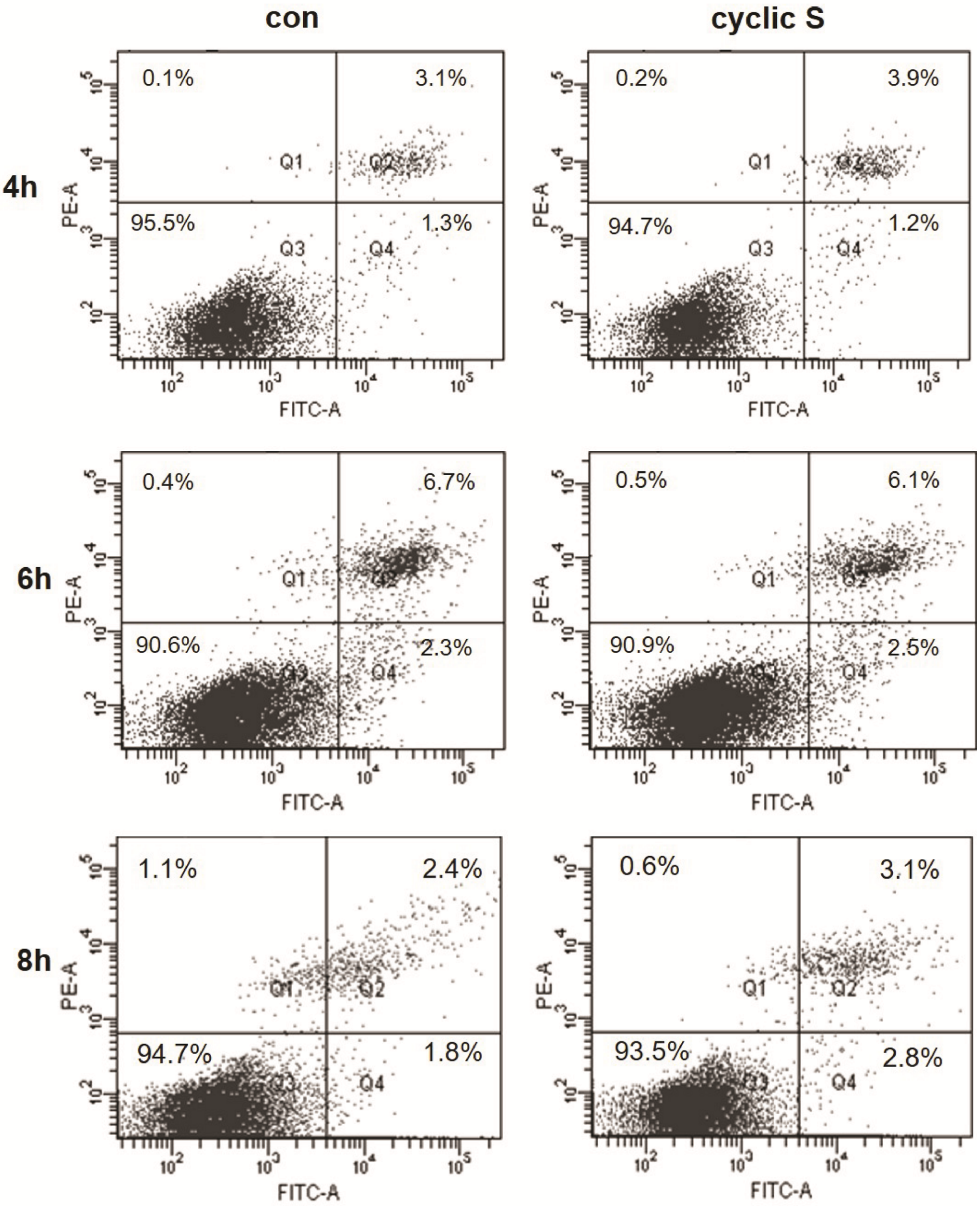
Cell viability during 4–8 h of cyclic stretch. MLE-12 cells were conditioned in serum free medium for 12 h and then transferred to wells in Bioflex and underwent cyclic stretch (cyclic S) or were not stretch (con) for 4, 6 or 8 h. Viable cells were negative for Annexin-V and propidium iodide (PI) staining (Q3), early-stage apoptotic cells were positive for Annexin-V staining, but negative for PI staining (Q4), and late-stage apoptotic cells were positive for Annexin-V and PI staining (Q2).

**Fig 2:**
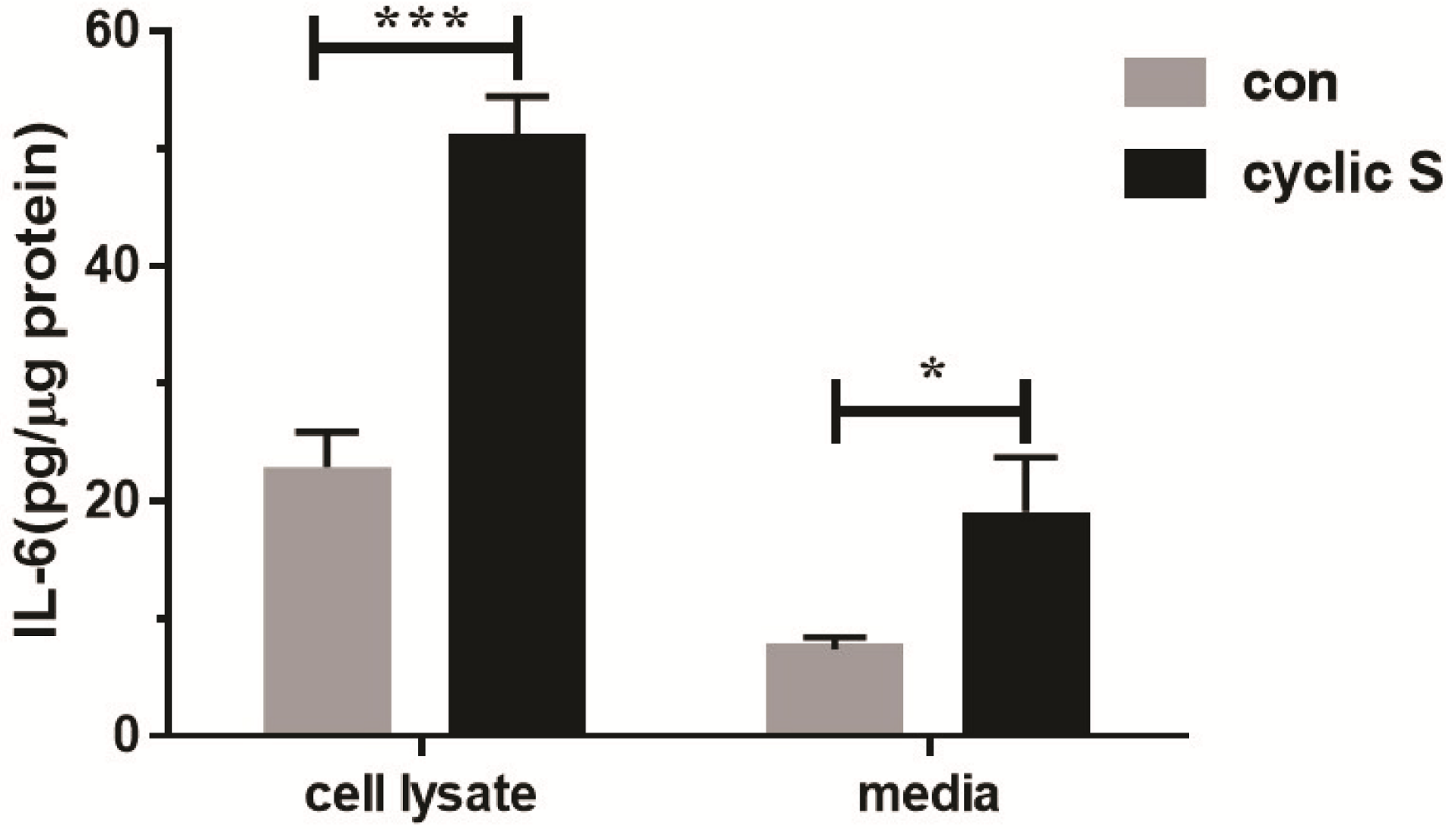
Cyclic stretch (6 h) induced increase in biosynthesis of IL-6. IL-6 levels in MLE-12 cell lysate and cell culture media with (cyclic S) or without stretch (con) were measured by ELISA. *P<0.05, ***P<0.001 compared with control. Each stretching group collected from three wells in a single experiment and the bar graphs illustrate data representative of three independent experiments.

Cell death was assessed by FACS with propidium iodide (necrosis) and annexin V (apoptosis) in MLE-12 that were conditioned in serum free medium for 12 h and then cyclic stretched (CS: ~18% elongation, 1 Hz) or not exposed to any stretch (con: controls). Cell viability remained at 91–96% over the 4–8 h experimental period and there were no differences in viability between CS and control suggesting that this magnitude of CS was not associated with cell death for MLE-12 (Fig 1). CS was however pro-inflammatory to MLE-12 cells as IL-6 levels in cell lysate or medium significantly increased at 6 h of stretch (Fig 2).

**Fig 3:**
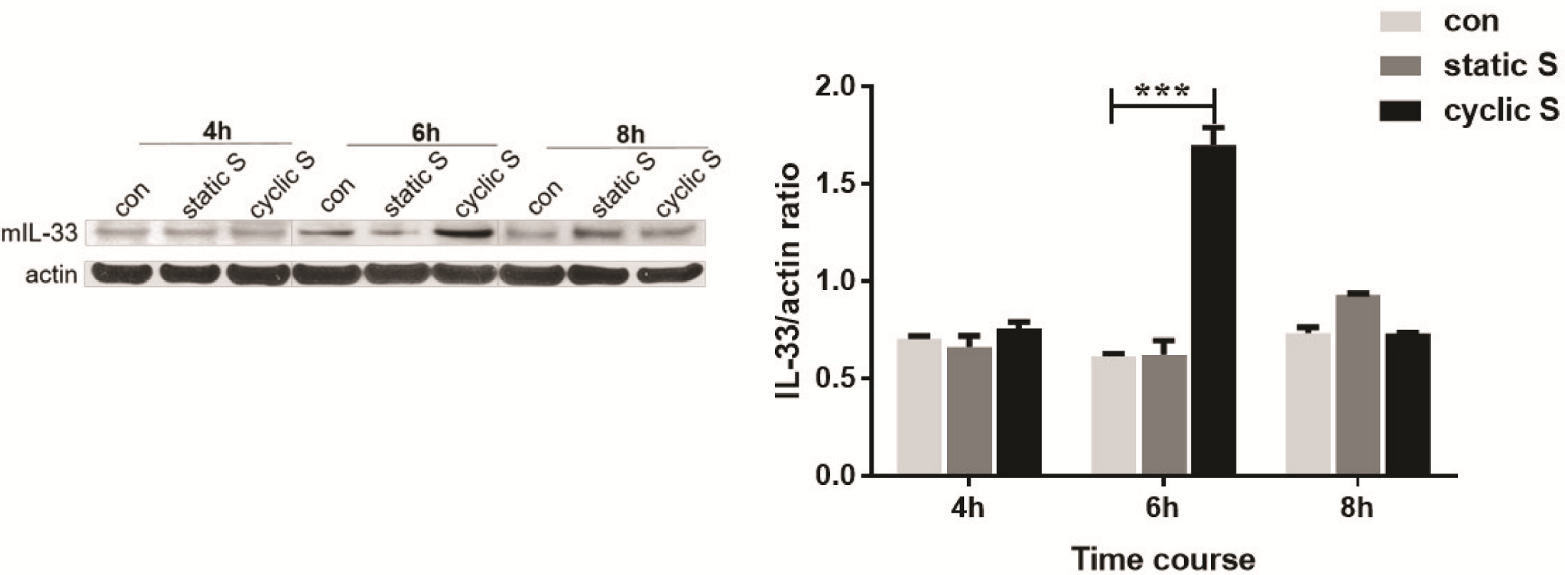
Cyclic, but not static, stretch induced increase in IL-33. Mouse IL-33 (mIL-33) expression in whole cell lysate after static (static S ~18% elongation) or cyclic stretch (cyctic S ~18% elongation, 1HZ) was detected by western blot at different time point (4h, 6h, 8h) and normalized by β-actin (right graph). ***P<0.001 compared with control (con). Each stretching group collected three wells for a single experiment, the bar graphs illustrate data representative of three independent experiments and western blot at the different time point was run separately.

**Fig 4:**
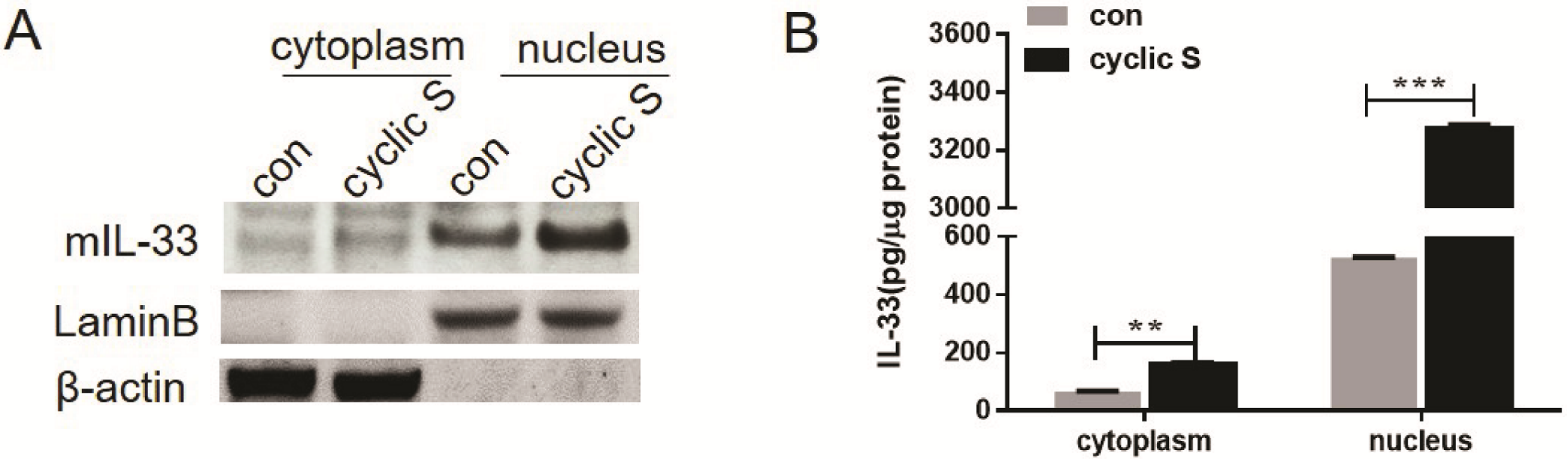
Cyclic stretch induced increase in cytosolic and nuclear levels of IL-33. Nuclear and cytoplasm protein fractions of MLE-12 cells with (cyclic S) or without (con) 6h stretch were isolated, and mIL-33 in nucleus and cytoplasm were measured by Western blot (A) or ELISA (B). Lamin B and β-actin (A) served as a loading control for nucleus and cytoplasmic protein, respectively. **P<0.01, ***P<0.001 compared with control (con). Each stretching group collected from three wells for a single experiment, the bar graphs illustrate data representative of three independent experiments.

We then contrasted the effect of cyclic vs. static stretch on cellular levels of IL-33. In Fig 3 we note a significant increase in whole cell levels of IL-33 at 6 h of CS that returned to control levels at 8 h. There were no significant changes in IL-33 in this interval in either control cells or those with static stretch. There was no detectable IL-33 in medium under any conditions. We further probed subcellular changes in IL-33 with CS by isolating cytoplasmic and nuclear fractions after 6 h of CS and measuring IL-33 by immunoblot (Fig 4A) and normalizing expression to subcellular markers (Lamin B for nucleus; beta actin for cytosol). IL-33 was detectable in both compartments and data from 3 separate co-cultures (Fig 4B) shows that CS significantly increased IL-33 in both compartments.

**Fig 5:**
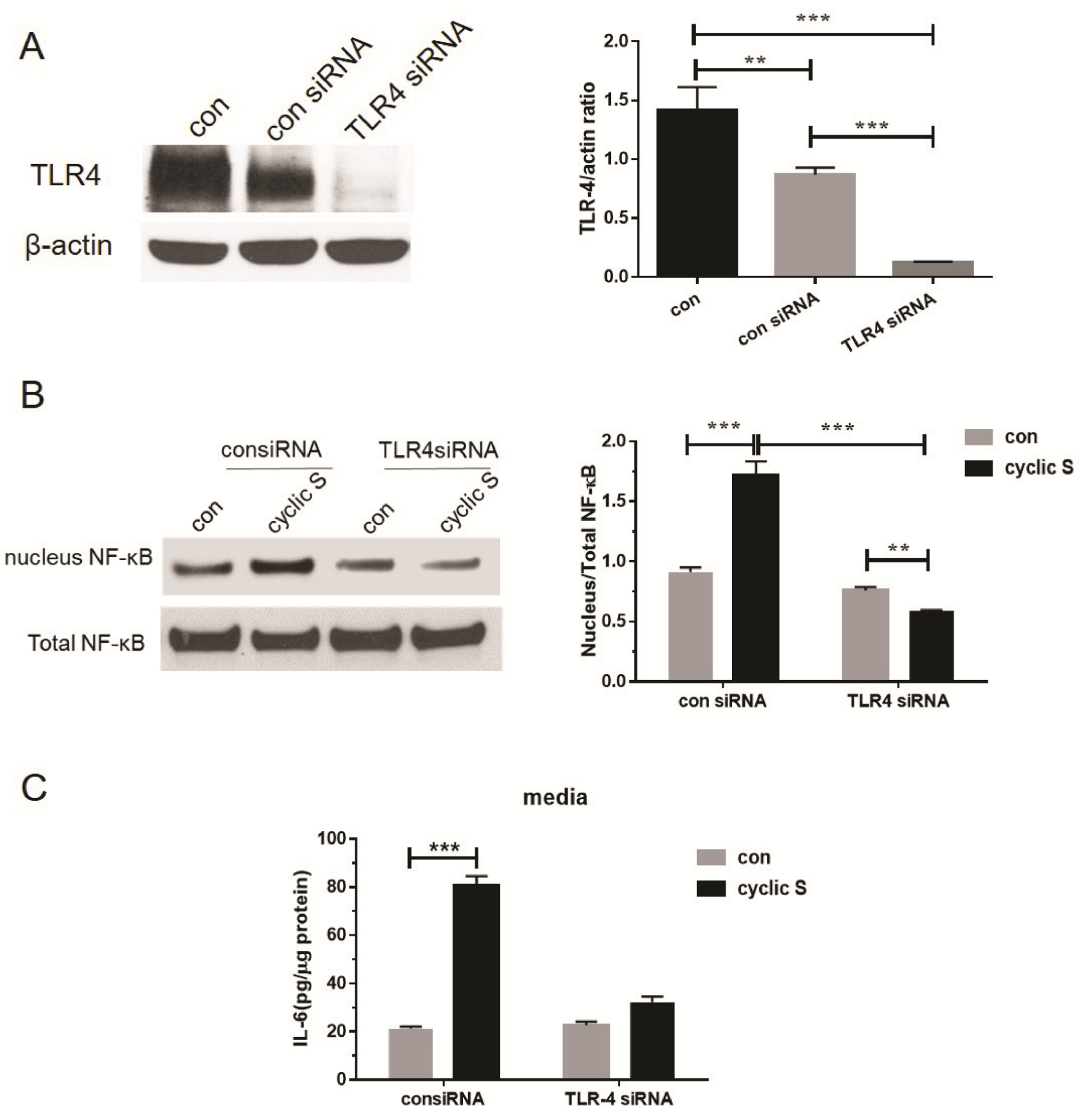
Cyclic stretch activates TLR4-dependent signaling (NF-κB translocation) and pro-inflammatory state (IL-6 release into medium). (A) MLE-12 cells were transfected with 50nM TLR-4 specific siRNA or negative control siRNA using Lipofectamine 2000 for 48 h. TLR-4 expression in control group (con), negative control siRNA group (con siRNA) and TLR-4 specific siRNA group (TLR4 siRNA) were detected by western blot and normalized by β-actin (upper graph). (B) Stretch induced NF-κB nucleus translocation were TLR-4 dependent. Total and nuclear NF-κB were measured by Western blot and its nucleus/total NF-κB ratio was analyzed after 6h cyclic stretch in transfected cells. (C) IL-6 secretion in transfected cell media after 6h cyclic stretch was measured by ELISA and was TLR-4 dependent. Each group collected from three wells in a single experiment and the data were presented as mean ± SD from three separate experiments. **P<0.01, ***P<0.001 when compared between groups denoted by horizontal lines.

We then sought to determine the role of TLR4 in stretch induced increases in IL-33 in MLE-12 cells. We first noted that MLE-12 cells express TLR4 and that targeted siRNA (but not scrambled or control siRNA) reduced TLR4 to barely detectable levels (Fig 5A). Activation of canonical pathway (e.g. translocation of NF-κB from cytosol to nucleus and release of pro-inflammatory cytokine, IL-6, to medium) was observed at 6 h of CS and this increase was sensitive to TLR4 ablation (Fig 5B and 5C, respectively).

**Fig 6:**
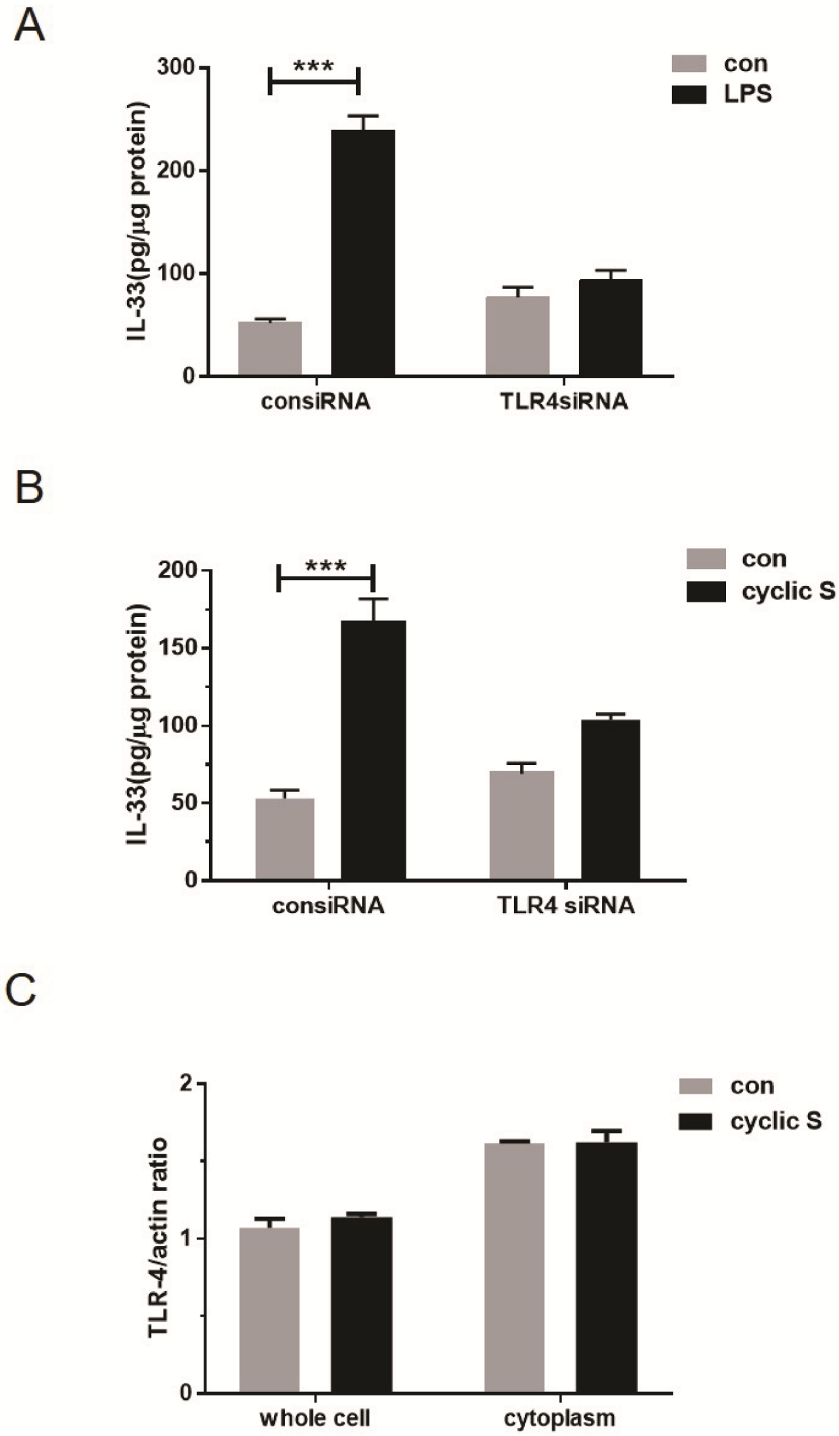
Activation of TLR4 by LPS or cyclic stretch increases intracellular IL-33. MLE-12 cells were transfected with 50 nM of TLR4-specific siRNA (TLR4siRNA) or nonspecific siRNA control (consiRNA) using Lipofectamine 2000 for 48h before stretch. (A) Transfected MLE-12 cells were further treated with 100 ng/ml LPS or control solution for 24h and IL-33 production were measured in cell lysate by ELISA. (B) IL-33 production after 6h cyclic stretch in transfected cells treated with or without TLR4-specific siRNA. (C) Total TLR4 expression in whole cell lysate and cytoplasm in MLE-12 cells with (cyclic S) or without (con) stretching were measured by Western blot and normalized by β-actin. ***P<0.001 when compared between groups denoted by horizontal lines.

To further confirm the role of TLR4 in mediating IL-33 biosynthesis, we contrasted the effect of the prototypic TLR4 agonist, LPS, to CS mediated effects in wildtype and TLR4 null cells. In Fig 6A we note that LPS increased cellular IL-33 in a TLR4 dependent fashion; in Fig 6B we note a similar TLR4 dependent mechanism for CS mediated IL-33 biosynthesis. Whole cellular or cytosolic levels of TLR4 were not affected by CS (Fig 6C).

### Role of HMGB1 in TLR4 mediated CS induced increase in IL-33 in MLE-12 cells

**Fig 7:**
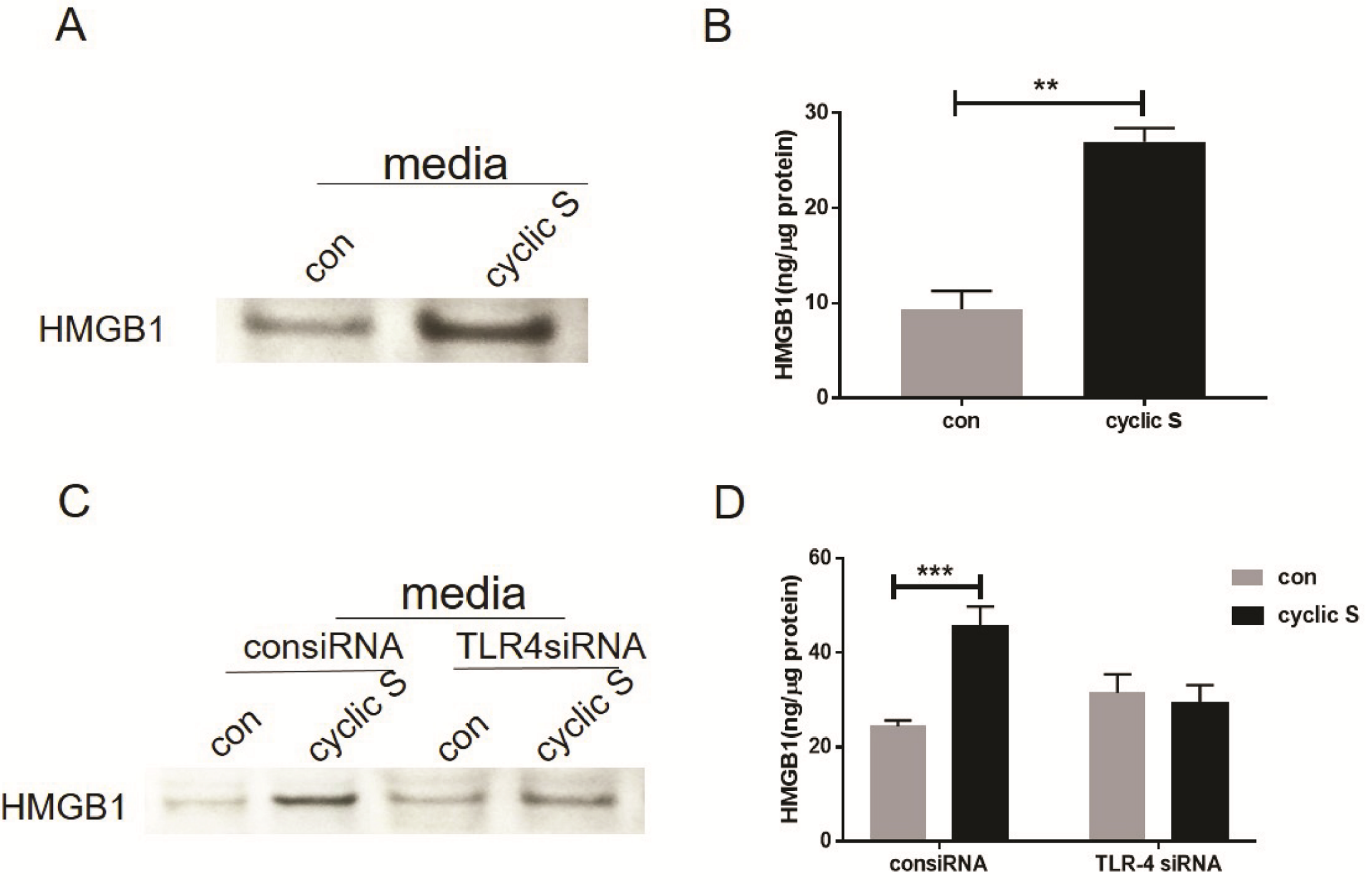
Cyclic stretch increases HMGB1 expression in a TLR4-dependent fashion. (A) Cell culture media with or without cyclic stretch were concentrated and HMGB1 contents were detected by Western blot. (B) HMGB1 in cell culture media with or without stretch was directly measured by ELISA. (C) Media of the cells which transfected with nonspecific siRNA control (consiRNA) or TLR-4-specific siRNA (TLR4 siRNA) with or without stretch (con vs. cyclic S) were concentrated and HMGB1 contents were measured by Western blot. (D) HMGB1 in media of the transfected cells following 6h cyclic stretch (cyclic S) was measured by ELISA. **P<0.01, ***P<0.001 compared with control. Each stretching group collected from three wells and represented a single experiment, the bar graphs illustrate data representative of three independent experiments.

**Fig 8:**
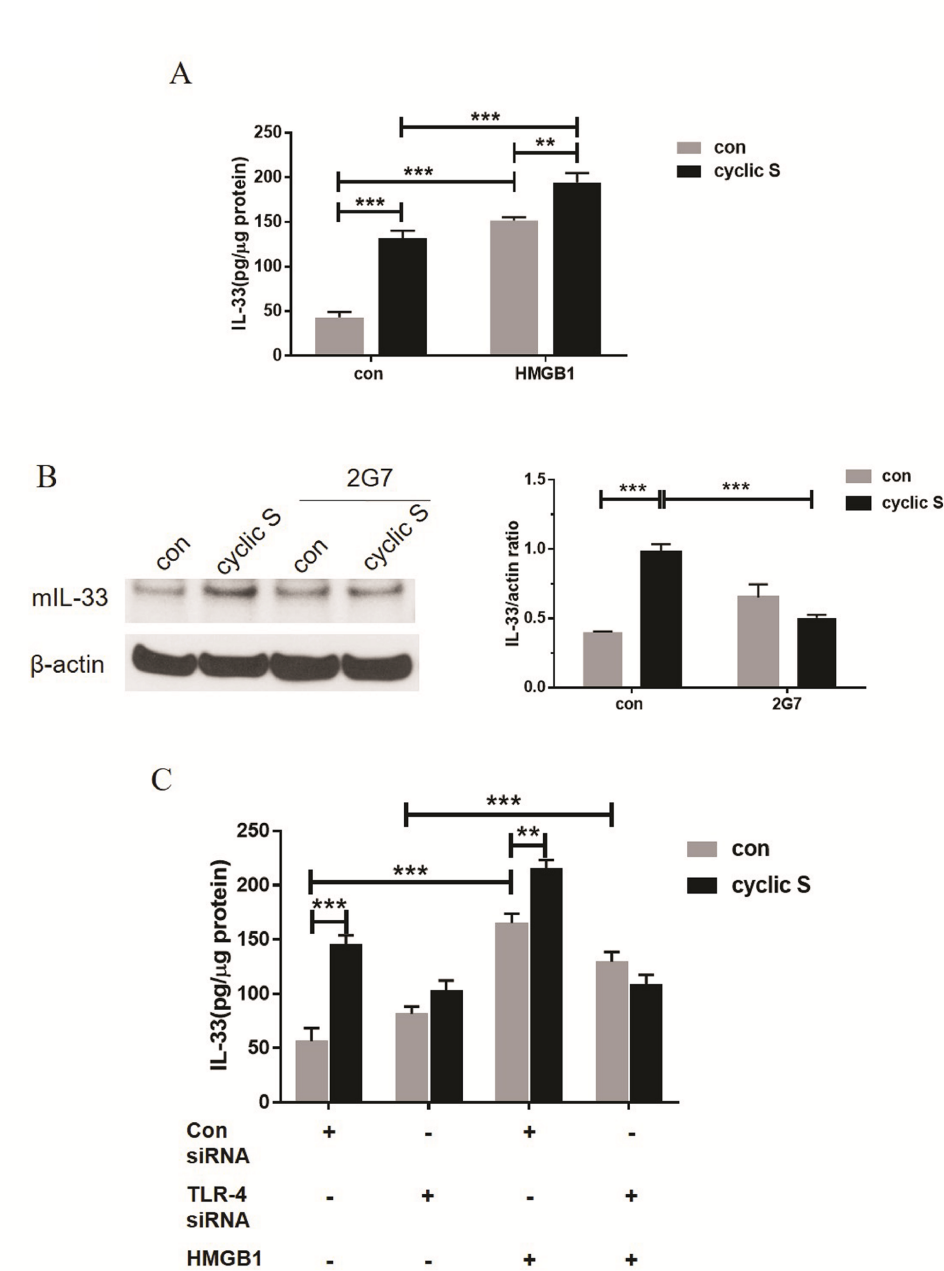
HMGB1 increases IL-33 expression and acts as an autocrine factor in enhancing IL-33 expression in a partial TLR-4 dependent fashion during cyclic stretch. (A) MLE-12 cells were treated with 3 μg/ml HMGB1 or control solution before 6h cyclic stretch and IL-33 production was measured by ELISA. (B) MLE-12 cells were treated with 10 μg/ml HMGB1 neutralizing antibody (2G7) or control solution before 6h cyclic stretch and IL-33 production was detected by Western blot. β-actin served as loading control. (C) MLE-12 cells transfecting with non-specific control siRNA or TLR-4-specific siRNA were treated with 3 μg/ml HMGB1 or control solution before stretch. IL-33 production in each group was measured with ELISA. **P<0.01, ***P<0.001 when compared between groups denoted by horizontal lines. Each stretching group collected at least from three wells and represented a single experiment, the bar graphs illustrate data representative of three independent experiments.

Since CS of human airway epithelium increased HMGB1 in an NF-κB fashion [23] and HMGB1 is an endogenous ligand of TLR-4 in airway epithelia cells [29–31], we hypothesized that HMGB1 may contribute to CS-TLR-4 mediated IL-33 biosynthesis. We noted an increase in immunoreactive HMGB1 in media of stretched MLE-12 cells by Western blot (Fig 7A) and ELISA (Fig 7B). Regardless of methodology to detect HMGB1, siRNA to TLR-4 blocked CS mediated increase in HMGB1 (Fig 7C and 7D). Exposure of MLE-12 cells to rHMGB1, alone, increased IL-33 in MLE-12 cells and CS significantly further increased IL-33 production due to HMGB1 (Fig 8A). A neutralizing antibody (2G7) to HMGB1 completely abrogated the CS mediated increase in IL-33 (Fig 8B). In cells treated with siRNA to TLR-4, rHMGB1 was still capable of increasing IL-33 (suggesting non-TLR4 mediated pathways for HMGB1) but rHMGB1 with CS did not increase IL-33 after siRNA to TLR4 in MLE-12 cells (Fig 8C).

## Discussion

In the current study, we stretched (~18% elongation) isolated cultured murine respiratory epithelial cells (MLE-12) on a flexible membrane in cyclic short term (4–8 h) fashion and noted a TLR4 dependent increase in intracellular IL-33 (Fig 6B) and extracellular HMGB1 (Fig 7). CS-induced increase in IL-33 was abrogated by neutralizing antibodies to HMGB1 (Fig 8) placing HMGB1 upstream of TLR4 mediated IL-33 biosynthesis but downstream of the undetermined stimulus by which stretch activates TLR4, itself. In this regard, HMGB1 is an autocrine factor acting on TLR4 in a positive feedback mode to cyclic stretch.

### CS and TLR4

We initially confirmed the report of Sebag et al [7] and showed that MLE-12 cells express TLR4 protein. siRNA to TLR4 decreased resting levels by more than 80% (Fig 5A). Stimulation of MLE-12 with LPS led to a TLR4 dependent increase in IL-33 (Fig 6B). CS increased IL-6 (Fig 2) in a TLR4 dependent fashion (Fig 5C) also consistent with a role for a functional TLR4 in MLE-12 [32, 33] as has been shown for LPS activated TLR4 and IL-6 secretion in human bronchial epithelial cells [34]. CS also caused nuclear translocation of NF-κB that was TLR4 dependent (Fig 5B). CS did not affect overall expression of TLR4 (as has been noted in stretched cardiomyocytes [6]) in our study (Fig 6C) similar to that noted by Kuhn et al [8] in primary cultures of rat alveolar type II cells but presumably caused increased surface expression of TLR4 as noted in MLE-12 cells by Sebag et al [7]; we did not combine LPS with stretch that led to decreased TLR4 surface expression and a reduction in release of keratinocyte derived cytokine (KC) and procoagulant tissue factor [7]. We did not pursue requisite roles for mCD14 in MLE-12 cells although others have noted mRNA for CD14 via in situ hybridization in mouse bronchiolar epithelium [35] and in primary bovine [36] and human [37] tracheobronchial epithelial cells. Transformed human bronchial epithelial cells (BEAS-2B) also express low amounts of CD14 on their surface but this is less clear in a number of other cultured human respiratory epithelial cells [38].

TLRs are pattern recognition receptors whose roles have expanded to include recognition of pathogen-associated molecular patterns in pathogens (such as LPS and TLR4) and endogenous ligands (see HMGB1 below) thereby contributing to both sterile injuries and non-infectious pathophysiology [39]. Mechanical stress is an important example of the latter and TLR4 plays a critical role in cardiac hypertrophy due to aortic banding and pressure overload [40] and VILI [2–5]. The ability to mimic mechanical stress by stretching isolated cells on a flexible membrane provides a useful experimental paradigm to minimize the multitude of factors that may converge on TLR4 in the intact animal. As such, the most compelling studies to date revealed an important role for TLR4 in CS stretch mediated sensitization of cardiac myocytes to TNF-α [6] and activation of the inflammasome in isolated alveolar macrophages [9]. Although highly relevant to the current study, Sebag et al [7] focused primarily on combined stretch with LPS exposure and associated down regulation of TLR4 with loss of LPS responsiveness. By focusing on CS and the alarmins, IL-33 and HMGB1, an additional pathway to CS and TLR4 was identified.

### CS and IL-33

Since its original discovery [10] as the functional ligand for ST2, an IL-1 receptor family member, IL-33 has been shown to act as a cytokine, transcriptional repressor, alarmin [11, 22] and a mechanically responsive cytokine in cardiomyocytes and fibroblasts [12, 13]. In addition to being expressed in some cells, such as macrophages and dendritic cells, IL-33 is also highly expressed in residential cells including epithelium of the upper [41] and lower [14] airways. In the lung, it has important roles in innate immunity and allergic lung inflammation [18], COPD [42], fibrosis [43] and acute lung injury [44, 45]. The current study was motivated in part on recent observations of an increase in immunoreactive IL-33 in the alveolar wall of mechanically ventilated rats [16]. It is of note that isolated type II cells from intact mice subjected to high tidal volume mechanical ventilation did not reveal an increase in IL-33 [17] suggesting the increase is perhaps restricted to type I cells in the alveolus or the response of IL-33 to stretch in situ is altered in the isolation and short term culture of type II cells. Although a TLR4-dependent IL-33 signaling pathway in allergic inflammation in mice was recently reported [18, 19], the link between IL-33 and TLR4 in non-infectious, non-allergic biosensing to mechanical stretch remains unclear.

In the current study, TLR4 is requisite for CS to increase levels of IL-33 in MLE-12 (Fig 6B). This was reinforced by the observation that LPS, the prototypical ligand for TLR4, also increased IL-33 and this was ablated in TLR4 siRNA treated cells (Fig 6A). Short term CS was not associated with secretion of IL-33 from MLE-12 cells, perhaps because there was no necrotic cell death (Fig 1) or because there were fundamental differences from that reported in fibroblasts [12, 13]. We did note an increase in both cytoplasmic and nuclear IL-33 after CS (Fig 4) but without performing more elegant biochemical studies of cellular localization [13], the directionality of nucleocytoplasmic translocation and other aspects of potential secretion were not apparent. Since we used short term CS that was not associated with cell death (Fig 1), it is possible that more intense or longer lasting CS may have led to necrosis and release of IL-33 to the extracellular space [46] as was noted by Yang et al [16] in which IL-33 was detected in bronchoalveolar lavage and plasma of intact mice with VILI or in the circulation of patients with ARDS or animals with experimental acute lung injury [45].

### CS and HMGB1/TLR4/IL-33 axis

We detected significant increases in HMGB1 in media conditioned from MLE-12 cells after cyclic stretch (Fig 7A and 7B) reminiscent of previous observations in A549 cells [23]. Secretion of HMGB1 was TLR4 dependent (Fig 7C) and was not passive as noted by lack of sufficient necrosis in CS (Fig 1) to account for this. HMGB1 is a member of HMG protein family and an abundant nonhistone nuclear protein that may be post-translationally modified and released from cells in response to a variety of stimuli [22]. Once released, HMGB1 mediates a number of biological functions including inflammation by binding to a number of surface receptors including TLR4 and receptor for advanced glycation end-products (RAGE). These pathways appear particularly important for the role of HMGB1 in sterile injury including mechanical injury [23]. We noted that siRNA to TLR4 partially antagonized (Fig 8C) rHMGB1-induced increased in IL-33 (Fig 8A) consistent with multiple receptors to transduce its effect. More importantly for the current study, neutralizing antibodies (2G7) to HMGB1 abolished the effect of CS on IL-33 biosynthesis (Fig 8B and 8C) suggesting that HMGB1 is upstream of CS induced TLR4 dependent increases in IL-33. A concept of an HMGB1/TLR4/IL-33 axis has been tested in other pathophysiological systems. Fu et al [47] showed that the release of HMGB1 is correlated with up-regulation of IL-33 in murine model of acute lung injury. An HMGB1-RAGE- and TLR4-dependent increase in experimental airway sensitization and inflammation [48] after house dust mite or cockroach sensitization was noted in mice. The most formal of an HMGB1/TLR4/IL-33 axis was recently [24] shown in diabetic cardiomyopathy in mice where high glucose mediated cardiomyocyte HMGB1 release interacts with TLR4 on cardiac fibroblasts and results in decrease in IL-33. Our results show that this potential paracrine/autocrine function of HMGB1 in response to stretch results in a TLR4-mediated increase in IL-33. Presumably the directionality (positive feedback in MLE-12 cells) of the effects are cell and tissue specific. As small molecules and neutralizing antibodies are available to antagonize each member of this HMGB1/TLR4/IL-33 pathway, it may be possible to purposefully manipulate components of stretch-induced changes in respiratory epithelium.
